# Exploring Perceptions and Practices Regarding Adult Vaccination against Seasonal Influenza, Tetanus, Pneumococcal Disease, Herpes Zoster and COVID-19: A Mixed-Methods Study in Greece

**DOI:** 10.3390/vaccines12010080

**Published:** 2024-01-12

**Authors:** Iordanis Avramidis, Ilias Pagkozidis, Philippe-Richard J. Domeyer, Georgios Papazisis, Ilias Tirodimos, Theodoros Dardavesis, Zoi Tsimtsiou

**Affiliations:** 1 Department of Hygiene, Social-Preventive Medicine and Medical Statistics, School of Medicine, Aristotle University of Thessaloniki, 54124 Thessaloniki, Greece; ioravramidis@gmail.com (I.A.); pagkoilias@auth.gr (I.P.); ityrodim@auth.gr (I.T.); dardaves@auth.gr (T.D.); 2 School of Social Sciences, Hellenic Open University, 26335 Patra, Greece; ph.domeyer@ac.eap.gr; 3 Department of Clinical Pharmacology, School of Medicine, Aristotle University of Thessaloniki, 54124 Thessaloniki, Greece; papazisg@auth.gr

**Keywords:** adult vaccination, vaccine hesitancy, influenza vaccine, pneumococcal vaccine, tetanus vaccine, herpes zoster vaccine, COVID-19, mixed methods, predictors, Greece

## Abstract

We aimed to document vaccination coverage for five vaccines, predictors of each vaccine’s uptake and attitudes regarding adult vaccination. Adults visiting four pharmacies were randomly invited to participate during summer 2022. Among 395 participants (mean age 51.2 years, range 19–96), vaccination rates were 78.1% for influenza and 25.8% for herpes zoster (≥60 years old), 64.3% for pneumococcal disease (≥65 years old), 33.1% for tetanus, while 11.4% had received two and 74.8% ≥3 COVID-19 vaccine doses. Half of participants (50.1%) voiced some degree of hesitancy, and 1.3% were refusers. The strongest predictor of each vaccine’s uptake was doctor’s recommendation (OR range 11.33–37.66, *p* < 0.001) and pharmacist’s recommendation (4.01–19.52, *p* < 0.05), except for the COVID-19 vaccine, where the Attitude Towards Adult VACcination (ATAVAC) value of adult vaccination subscale’s score was the only predictor (OR: 5.75, *p* < 0.001). Regarding insufficient coverage, thematic content analysis revealed seven main themes. Insufficient knowledge, the absence of health professionals’ recommendation, perception of low susceptibility to disease, negligence and dispute of vaccine effectiveness were universal themes, whereas safety concerns and distrust in authorities were reported solely for COVID-19 vaccination. Designing public interventions aiming to increase trust in adult vaccination is essential in the aftermath of the COVID-19 pandemic. Health professionals’ role in recommending strongly adult vaccination is crucial.

## 1. Introduction

Vaccination is among the greatest, most successful, and cost-effective public health interventions [1,2,3]. Immunization policies and practices have aided in curbing and eradicating diseases, currently preventing two to three million deaths from vaccine-preventable diseases [2]. Contrary to high coverage rates in the pediatric population, vaccine coverage in adults is poor, with thousands of deaths and increasing healthcare costs being annually attributed to vaccine-preventable diseases [1].

With pivotal importance, immunization-related hesitancy is gaining ground and is considered one of the ten major threats to public health [2]. Vaccine hesitancy describes the refusal or deferment of vaccines despite the availability of such services. It is a complex, behavioral phenomenon that is context specific and varies across time, place and vaccine type [4,5]. As the public’s attitudes towards vaccines are represented on a continuum, ranging from complete agreement to total refusal, those hesitant towards vaccination represent a rather heterogenous group that may accept all vaccines with caution; may accept some, yet reject others; may outright refute vaccines or even be unsure in proceeding with vaccination [5,6].

Few adults are protected against all vaccine-preventable diseases, as shown by national adult vaccination programs [7], and coverage in high-income countries remains suboptimal [7,8,9,10]. Inadequate coverage for pneumococcal disease, herpes zoster and tetanus is highlighted among older Greeks, whereas influenza coverage is high [11]. Yet, a study commissioned after the COVID-19 pandemic underscored the unwillingness to undergo influenza vaccination, with only 39.4% of citizens eager to vaccinate [12]. During the pandemic, mistrust of vaccine safety, effectiveness, governments and political decisions, low risk perception and the spread of fake news had an important role in the development of hesitancy towards the novel vaccines [13]. Over a quarter of the European Union population was reluctant about COVID-19 vaccination, with Bulgaria showing the highest hesitancy rates (61%) [14]. Moreover, hesitancy to undergo booster vaccination and doubts regarding its effectiveness and necessity were recorded [15]. In the literature, adult vaccine hesitancy has been associated with anxiety, fear, low perception of susceptibility, the absence of health professionals’ recommendation, previous negative experience, mistrust of pharmaceutical companies and vaccine effectiveness, negligence, access barriers, as well as lack of information, resulting in suboptimal vaccination rates [16].

As we transition to the post-COVID era, documenting vaccine coverage and identifying and understanding the barriers to adult immunization are pivotal in shaping effective vaccination programs, whilst developing strategies to address hesitancy amongst the general population [17]. To this end, this study is the first internationally to investigate the perceptions regarding adult vaccination in the aftermath of the pandemic, as well as the vaccination coverage against influenza, tetanus, pneumococcal disease, herpes zoster and COVID-19 and the predictors of their uptake. Recognizing the differences in attitudes and practices regarding adult immunization, it aids in detecting the barriers to be overcome effectively in pursuit of universal vaccination coverage for adults, according to national adult vaccination programs, in light of the new challenges highlighted by the recent pandemic.

## 2. Materials and Methods

### 2.1. Study Design and Setting

A multicenter, mixed-methods design was utilized to record vaccination coverage and related perceptions among adults attending four pharmacies in the Municipality of Amyntaio, Florina Prefecture, Greece. Three pharmacies were in the city of Amyntaio (4318 inhabitants, 2021 census) and one in the village of Levaia (660 inhabitants, 2021 census). The survey was conducted during the period July–September 2022.

### 2.2. Study Population and Sampling

Adults attending four pharmacies in the Municipality of Amyntaio were invited to participate in the study. The sample size was determined at 377 participants, allowing for a 95% confidence interval (CI) and a 0.05 margin of error, according to the normal Gaussian distribution. Based on the 75.9% response rate reported in a recent Greek multicenter study [16], a total number of 502 adults were to be approached with the help of a random number generator. Participation was anonymous and optional, and eligible participants were required to sign a consent form. Those under 18 years old, as well as those with severe mental and/or physical conditions hindering the appropriate completion of the study tool, were excluded. To minimize social desirability bias, participants returned the study tool in two opaque ballot boxes on pharmacy premises.

### 2.3. Study Tool

The study tool consisted of three distinct parts. Participants first completed a sociodemographic survey, documenting the age, sex, employment, educational level, marital status, and presence of children. The study tool was pilot tested to evaluate understanding in a convenience sample of 20 individuals.

The second part explored their perceptions regarding adult vaccination. They were particularly asked to identify themselves across the five categories of the vaccine hesitancy continuum: (a) accept all adult vaccines; (b) accept adult vaccines, but unsure; (c) accept some adult vaccines, but delay or refuse some; (d) refuse adult vaccines, but unsure; and (e) refuse all adult vaccines [5]. Participants then completed the valid and reliable Attitude Towards Adults Vaccination (ATAVAC) scale [18]. The ATAVAC scale consists of 11 items, in which participants respond with their degree of agreement using a 6-point Likert scale (ranging from 1 = strongly disagree to 6 = strongly agree), and three subscales: (a) “Value of adult vaccination” (7 items); (b) “Safety concerns” (2 items); and (c) “Perceived barriers” (2 items). Participants’ scores range from 1 to 6, with higher scores indicating more positive attitudes towards adult vaccination. Higher scores in the “Value of adult vaccination” subscale indicate favorable perceptions regarding the value of adult immunization, whereas higher scores in the “Safety concerns” and the “Perceived barriers” subscales indicate fewer concerns regarding vaccine safety and fewer practical barriers to vaccinations, respectively.

The third part included questions about immunization practices. History of adult vaccination was inquired about, alongside vaccination status for four diseases, as indicated by the National Immunization Program for Adults 2022, and COVID-19. Specifically, participants were asked whether they got vaccinated against seasonal flu (during the 2021–2022 season for people ≥60 years old or younger adults who belong to well-defined high-risk groups), tetanus (at least once within the last 10 years), pneumococcal disease (at least once for people ≥65 years old or younger adults who belong to well-defined high-risk groups) and herpes zoster (vaccinated with the single-dose live zoster vaccine for people 60–75 years old). Additionally, they were asked about the number of doses of COVID-19 vaccine they had, ranging from zero (unvaccinated), one (partially vaccinated), two (fully vaccinated/completed primary COVID-19 vaccination series of two doses) to three or more shots (having received booster doses). Regarding each vaccine, participants were asked to elaborate on: a. the reason for no immunization (open-ended question); b. their level of information about the specific vaccine using a 10-point Likert scale (ranging from 0 = not informed at all to 10 = outstandingly informed); c. health professionals’ recommendation (dichotomous answer, yes/no); and d. the actors influencing their opinion on the specific vaccine (multiple answer question: “Family physician’s recommendation”, “Specialist’s recommendation”, “Pharmacist’s recommendation”, “Friends and family”, “Mass media”, “Internet sources”). Participants were asked about the number of COVID-19 vaccine doses administered by the time of study tool completion (ranging from zero to four), as well as the reason for attending the pharmacy. Personal medical history was also inquired about to record conditions that place adults in high-risk groups, thus defining the necessity of carrying out adult vaccinations. Ultimately, they were asked to determine whether they would like to be better informed about the vaccines included in the National Adult Immunization Program (dichotomous response, yes/no).

### 2.4. Data Analysis

Data analysis was performed with Jamovi (Version 2.3, 2023), free and open statistical software. The Shapiro–Wilk test examined whether continuous variables were distributed normally. Those with a normal distribution are presented as mean (M) and standard deviation (SD), while non-parametric ones with median and interquartile range (IQR: Q1, Q3). Qualitative variables are presented as absolute values and percentages of total responses per question—n (%). To compare normally distributed scores, a one-way ANOVA and chi-squared test were performed. Logistic regression analyses were performed to examine possible predictors influencing the uptake of each of the five vaccines after controlling for sociodemographic variables. The latter included age, sex, low educational level (defined as ≤6 years), marital status (married vs. other) and smoking status. Independent variables with statistically significant correlations in the univariate analyses were included in the multivariate models. Significance level was set at 0.05 and two sided.

To analyze qualitative data, provided by participants’ answers to the open-ended question regarding the possible reasons for not getting vaccinated against each one of the studied diseases, thematic content analysis was conducted, up to reaching data saturation [19]. Data analysis was performed independently by two authors, one nurse and one physician, health professionals with training and experience in qualitative data analysis. The researchers began with open coding of participants’ responses. They developed, agreed upon, applied, and iteratively refined a coding framework, where the open codes were refined into the seven major themes.

## 3. Results

A total of 395 individuals participated in the study (78.7% response rate). The sociodemographic characteristics of participants are illustrated in Table 1, whereas information on medical history, smoking status, and reason for attending the pharmacy are presented in Table 2. Mean age of participants was 51.2 years (SD = 17.1, min. 19–max. 96). A known diagnosis of at least one chronic disease was declared by 138 individuals (34.9%).

### 3.1. Attitudes towards Adult Vaccination

With regard to participants’ stances towards adult immunization, 48.6% (190/391) were in favor of all adult vaccines, half of them (196/391, 50.1%) voiced some degree of hesitancy (“in favor of adult vaccines, but not sure about all”: 38.6%, “in favor of some adult vaccines, but not sure about all”: 9.7%, “against adult vaccines, but not sure about all”: 1.8%), while vaccine refusers corresponded to 1.3% (5/391). The vast majority had at least one vaccination as an adult (363/395, 91.9%), and 84.1% (332/395) would like to be better informed about the recommended vaccines for their age and health status, according to the National Adult Immunization Program.

Participants’ mean score on the ATAVAC scale was 4.72 (SD = 0.71, min. 1.90–max. 5.81). Their subscale scores were: (a) Value of adult vaccination (4.86, SD = 0.80, min. 1.1–max. 6); (b) Safety concerns (3.11, SD = 1.38, min. 1–max. 6); and (c) Perceived barriers (5.36, SD = 0.89, min. 1–max. 6). Scores on the ATAVAC scale and respective subscales according to participant’s responses in the continuum of vaccine hesitancy are illustrated in Table 3, while ATAVAC scores per item are presented in Table 4.

### 3.2. Coverage, Degree and Source of Information Regarding Vaccines

Vaccination coverage for seasonal influenza, tetanus, pneumococcal disease, herpes zoster and COVID-19 among participants is highlighted in Table 5, while sources of influence regarding each vaccine are illustrated in Table 6. The main reasons guiding participants’ decisions to not vaccinate themselves, as derived from thematic content analysis, as well as illustrative quotes per theme, are presented in Table 7.

#### 3.2.1. Influenza Vaccination

In our study, 48% of participants (189) had received a flu shot in the past year, 100 of which (52.9%) met the age criterion defined by the National Adult Immunization Program (≥60 years old), whereas remaining ones belonged to different high-risk groups. In total, 78.1% (100/128) of participants ≥60 years old were vaccinated according to the National Adult Immunization Program. Vaccination practices increased with age (*p* < 0.001) and were not influenced by participants’ sex (*p* = 0.19) (Table 5). Participants were well informed about seasonal influenza vaccines (median of 7, IQR = 5, 8.3 on a 10-point Likert scale), while a quarter of participants reported insufficient knowledge about the vaccine (99/394, 25.1%) (Table 6). Doctors were the main source of information, with family physicians influencing participants’ views about influenza vaccine in 38.8%, followed by specialists in 15% of participants (Table 6). The multivariate logistic regression revealed that the predictors for influenza vaccine uptake were increasing age (OR: 1.04; 95% confidence interval (CI): 1.01–1.06; *p* = 0.017), suffering from a chronic disease (OR: 2.77; 95% CI: 1.34–5.70; *p* = 0.006), increased trust in the value of adult vaccination (ATAVAC subscale score, OR: 2.45; 95% CI: 1.50–4.01; *p* < 0.001), as well as vaccination recommendation from their doctor, either family physician or specialist (OR: 11.33; 95% CI: 5.86–21.90; *p* < 0.001), and their pharmacist (OR: 5.25; 95% CI: 1.79–15.42; *p* = 0.003) (Table 8).

Regarding insufficient vaccination coverage against influenza, thematic content analysis of the qualitative data in the relevant open-ended question revealed four main themes. Those abstaining from influenza vaccination reported that reasons for not getting vaccinated against this disease were: the absence of health professionals’ recommendations, a perception of low susceptibility to the flu, doubts over vaccine effectiveness and negligence. Illustrative quotes of each theme are presented in Table 7.

#### 3.2.2. Tetanus Vaccination

In the past decade, only 33.1% (130) of participants were vaccinated against tetanus (Table 5). In our study, age and sex correlated significantly with vaccination practices, with men (*p* < 0.001) and younger participants (*p* = 0.016) being mostly immunized. Participants were moderately informed (median of 5, IQR = 2, 7 on a 10-point Likert scale), with 42% of participants reporting insufficient knowledge (Table 6). The multivariate logistic regression revealed that the predictors for tetanus vaccine uptake were younger age (OR: 0.97; 95% CI: 0.95–0.99; *p* = 0.044), male sex (OR: 2.79; 95% CI: 1.50–5.19; *p* = 0.001), increased ATAVAC perceived barriers subscale score (OR: 1.54; 95% CI: 1.03–2.32; *p* = 0.036) as well as vaccination recommendation from their doctor, either family physician or specialist (OR: 22.82; 95% CI: 11.80–44.14; *p* < 0.001), and their pharmacist (OR: 4.01; 95% CI: 1.13–14.24; *p* = 0.032) (Table 8).

Regarding insufficient vaccination coverage against tetanus, thematic content analysis of the qualitative data in the relevant open-ended question revealed five main themes. Those abstaining from tetanus vaccination reported that reasons for not getting vaccinated against this disease were: the absence of health professionals’ recommendations, the lack of information regarding disease and the vaccine, low perceived vulnerability, doubts over vaccine effectiveness and negligence. Illustrative quotes of each theme are presented in Table 7.

#### 3.2.3. Pneumococcal Vaccination

In total, 26.6% (105/394) of participants had been vaccinated against pneumococcus. Of those, 62 (59%) had received one and 43 (41%) had received all recommended vaccines. Out of 105 vaccinated participants, only 84 (79.3%) met the age criterion (≥65 years old) according to the National Adult Immunization Program, with 64.3% (54/84) being vaccinated at least once. Pneumococcal vaccination increased with age (*p* < 0.001) and was higher in women (*p* = 0.035) (Table 5).

Participants’ level of information was moderate (median of 5, IQR = 2, 7 on a 10-point Likert scale), with 44% (173/393) of respondents reporting insufficient knowledge to decide upon the pneumococcal vaccine (Table 6). The multivariate logistic regression revealed that the predictors for pneumococcal vaccine uptake were increasing age (OR: 1.05; 95% CI: 1.01–1.08; *p* = 0.005), the presence of a chronic disease (OR: 3.03; 95% CI: 1.21–7.57; *p* = 0.018), as well as vaccination recommendation from their doctor, either family physician or specialist (OR: 32.41; 95% CI: 13.63–77.08; *p* < 0.001), and their pharmacist (OR: 8.43; 95% CI: 2.21–32.11; *p* = 0.002) (Table **8**).

Regarding insufficient vaccination coverage against pneumococcus, thematic content analysis of the qualitative data in the relevant open-ended question revealed five main themes. Those abstaining from pneumococcal vaccination reported that reasons for not getting vaccinated against this disease were: insufficient information about the vaccine, the absence of health professionals’ recommendations, negligence, doubts over vaccine effectiveness and low subjective sense of vulnerability. Illustrative quotes of each theme are presented in Table 7.

#### 3.2.4. Herpes Zoster Vaccination

In total, 25.8% of the participants ≥60 years old were vaccinated against herpes zoster. Coverage increased with age (*p* < 0.001) and was not related to gender (*p* = 0.31) (Table 5). The median value of vaccine awareness self-assessment was 5 (IQR = 2, 7, 10-point Likert scale), with 42.8% (169/395) of respondents reporting insufficient knowledge to decide upon the herpes zoster vaccine (Table 6). The multivariate logistic regression revealed that the predictors for herpes zoster vaccine uptake were increasing age (OR: 1.05; 95% CI: 1.00–1.10; *p* = 0.043), marital status other than being married (OR: 0.16; 95% CI: 0.05–0.56; *p* = 0.004), the presence of a chronic disease (OR: 9.32; 95% CI: 1.77–49.11; *p* = 0.008), not being a current smoker (OR: 0.15; 95% CI: 0.03–0.69; *p* = 0.015), increased trust in the value of adult vaccination (ATAVAC subscale score, OR: 3.24; 95% CI: 1.31–8.04; *p* = 0.011), as well as vaccination recommendation from their doctor, family physician or specialist (OR: 37.66; 95% CI: 10.02–141.56; *p* < 0.001), and their pharmacist (OR: 19.52; 95% CI: 3.11–122.72; *p* = 0.002) (Table 8).

Regarding insufficient vaccination coverage against herpes zoster, thematic content analysis of the qualitative data in the relevant open-ended question revealed four main themes. Those abstaining from herpes zoster vaccination reported that reasons for not getting vaccinated against this disease were: the absence of health professionals’ recommendations, inadequate information, low sense of susceptibility and negligence. Illustrative quotes of each theme are presented in Table 7.

#### 3.2.5. COVID-19 Vaccination

Overall, 11.9% (47/394) of participants were not vaccinated, and 1.8% (7/394) were partially vaccinated (one dose). A primary vaccination course with two doses was completed by 11.4% (45/394) participants, whereas 74.8% (295/394) had three to four booster doses. Vaccination coverage increased with age (*p* < 0.001) and was no different between sexes (*p* = 0.09) (Table 5). Participants were highly informed about COVID-19 vaccine (median of 8, IQR = 7, 9 on a 10-point Likert scale), and only 1.8% (7/395) reported insufficient knowledge (Table 6). The multivariate logistic regression revealed that the only predictor for COVID-19 vaccine uptake was the increased trust in the value of adult vaccination, as described by the ATAVAC value of adult vaccination subscale’s score (OR: 5.75; 95% CI: 3.03–10.93; *p* < 0.001) (Table 8).

Regarding insufficient vaccination coverage against COVID-19, thematic content analysis of the qualitative data in the relevant open-ended question revealed four main themes. Low sense of vulnerability and doubts over vaccine efficacy were the main reasons behind vaccination refusal according to qualitative analysis. Moreover, fear of adverse effects and participants’ reaction to obligatory vaccination/questioning of institutions’ motives were the main themes reported solely for the COVID-19 vaccines. Illustrative quotes of each theme are presented in Table 7.

## 4. Discussion

### 4.1. Main Findings

In this mixed-methods study, we investigated vaccination coverage for four diseases, as indicated by the National Adult Immunization Program, and COVID-19, in the context of the pandemic, as well as perceptions toward adult vaccination in a random sample of adults visiting four pharmacies in Greece. Vaccination coverage for COVID-19 and influenza was reportedly high among high-risk groups, while only two-thirds of those in high-risk groups for pneumococcal disease were vaccinated. Coverage rates for tetanus and herpes zoster were the lowest. This study also highlighted the predictors influencing adult vaccination rates, including sociodemographic factors, health status factors, ATAVAC subscale scores and vaccination recommendations from doctors and pharmacists.

Nearly half of participants were in favor of all adult vaccines. A significant proportion was supportive of immunization, holding a few reservations regarding some vaccines, providing a better picture of the continuum of vaccine hesitancy in the Greek population. Moreover, participants’ scores on the ATAVAC scale were on the positive side (4.72 out of 6, SD = 0.71). Participants viewed adult vaccination favorably (4.86 out of 6, SD = 0.80) and perceived fewer practical barriers to immunization (5.36 out of 6, SD = 0.89). However, they expressed a medium level of concern over vaccine safety (3.11 out of 6, SD = 1.38). ATAVAC scores corresponded to participants’ self-reported overall views towards vaccines.

Recommendations from health professionals, especially doctors, either family physicians or specialists, were pivotal in acceptance of all studied vaccines and emerged as the strongest predictor for all vaccines’ uptake, except for COVID-19. Acknowledging the value of adult vaccination was a strong predictor for influenza and herpes zoster vaccine uptake and the only predictor for COVID-19 vaccines, while the perceived barriers in adult vaccination seemed to predict tetanus vaccine uptake. Moreover, inadequate knowledge about the tetanus, pneumococcal and influenza vaccines was highlighted. Most participants call for more information about the recommended vaccines for their age and health status according to the National Adult Immunization Program.

Seven main arguments about vaccination refusal emerged through thematic content analysis for the studied vaccines: a. absence of health professionals’ recommendation; b. perception of low susceptibility to disease; c. dispute of vaccine effectiveness; d. negligence; e. insufficient information (regarding disease and/or vaccine); f. fear of side effects; and g. distrust in authorities/opposition to obligatory vaccination. The two last themes were reported solely for COVID-19 vaccination.

### 4.2. Comparison with Existing Literature

A recent study among elderly Greeks, over 60 years old, reported high coverage for influenza (83%), moderate coverage for pneumococcal disease (49.7% for the conjugate and 23.2% for the polysaccharide vaccine) and poor coverage for herpes zoster (20.7%) and tetanus (7.3%) [11]. In a like manner, in a nation-wide sample just before the pandemic similar vaccine coverage percentages were reported for influenza (55%), pneumococcal disease (36%) and tetanus (21%) [20]. Coverage in our study follows a similar pattern, with the highest rates for seasonal influenza, moderate ones for pneumococcal disease and poor immunization for tetanus and herpes zoster. Influenza coverage in our participants was higher compared to EU (50.8%, 2021) and US (69.7%, 2022) rates, and well above the World Health Organization’s target (75%) [21,22,23]. Vaccination against pneumococcal disease is suboptimal in Europe (17.95% of clinical-risk groups), a finding reflected in our study [24]. Our elder participants reported similar coverage to Australian, English and US counterparts [25,26,27]. Yet, our rate (64.3%) was higher than the European average (24.2% of ≥65 years old) [24], which may be attributed to high-risk participants visiting pharmacies being more likely to be influenced positively about the vaccine. Coverage for tetanus varied greatly between countries, with a study among six European countries underscoring low rates in Italy, Poland and Greece [28]. Associations of coverage with demographic characteristics were similar with a previous study in terms of age (influenza, pneumococcus, tetanus) and sex (influenza, herpes zoster, tetanus), whereas in our sample pneumococcal vaccination was more common in women and herpes zoster in older adults [20]. As far as COVID-19 vaccination is concerned, the overwhelming majority of participants (86.2%) had at least two doses of the primary course, a finding comparable to the European (82.4%) and Greek vaccination profiles in adults over 18 years old (82.5%) [29].

Study participants were confident about adult vaccination, as reflected in ATAVAC scores. Lower scores, implying skepticism, were recorded by those self-reporting negative attitudes towards adult immunization, a finding also reported with other scales estimating adult vaccine confidence. In an Israeli study, pro-vaccine participants showed affirming stances through lower scores in the Vaccine Attitudes Examination Scale (VAX) [30]. Higher VAX scores, and thus lower trust in the value of adult vaccination, were similarly reported among Turkish refusers, those not intending to vaccinate against COVID-19, and those without prior adult vaccination history [31]. Vaccine acceptance also correlated positively with confidence in COVID-19 vaccination, as measured with the Vaccine Hesitancy Scale in Singapore [32]. Going forward, utilizing scales that go beyond trust and confidence, such as the ATAVAC scale, will be crucial in quantitatively examining vaccine hesitancy and its determinants across different populations.

Doctors, and especially family physicians, were the primary source of information for all vaccines, influencing participants’ views on immunization and vaccination uptake, followed by pharmacists, reiterating findings of previous Greek, Dutch and Spanish studies [20,33,34,35]. Recent US surveys showcased the public’s trust in doctors, especially primary care practitioners, as a source of reliable information regarding vaccines [36,37]. Except for influenza and COVID-19, for which participants self-reported strong understanding, family physicians could utilize long-standing trust and motivational interviewing to mitigate fears, increase awareness and primary prevention [38] and restore declining vaccine confidence in the post-pandemic era [39], joining forces with specialists. Pharmacists, as more accessible health providers, were highly trusted, and could play a significant role in vaccine promotion and uptake, ensuring their safe and effective administration. They could be key actors in preventive medicine and their omnipresence across community settings, the reduced cost and extended hours of operation could facilitate efforts to increase coverage and tackle hesitancy [40]. Additionally, participants underscored the role of mass media and the internet in disseminating vaccine information. Yet, these have mostly been associated with providing disinformation and deliberate spreading of fake news [41], especially through refusers’ alarming anti-vaccine footprint on social media [42]. This was reflected in a previous Greek study, where being informed by social media predicted self-reported hesitancy and increased focus on vaccine side effects [33].

When examining predictors influencing uptake patterns for the studied vaccines, motivation to get vaccinated based on doctors’ and pharmacists’ recommendations was the strongest and most consistent predictor for all but the COVID-19 vaccines. These findings corroborate our previous study [20] and further highlight the importance of healthcare professionals in reinforcing vaccination uptake. On the other hand, mass media campaigns did not seem effective enough in promoting vaccination uptake. In addition, the attitudes towards adult vaccination, as expressed by the belief in the value of adult vaccination, were associated with the uptake of all studied vaccines, except for the pneumococcal and tetanus vaccines. The pivotal role of attitudes towards adult vaccination in determining vaccination uptake has already been highlighted [20] and, therefore, should be a key target of public health initiatives. In addition, the presence of comorbidities was also associated with receiving influenza, pneumococcal and herpes zoster vaccines, probably reflecting specific targeting by their physicians or more frequent healthcare visits, as already stressed in our previous work [20]. Male gender only seemed to influence the uptake of tetanus vaccine. Age was a strong predictor of influenza, pneumococcal and herpes zoster vaccine uptake, perhaps reflecting the impact of the National Adult Immunization Program, in which these vaccines are mainly recommended for older adults.

Delving into participants’ arguments about abstinence, thematic content analysis revealed themes that fit the proposed “3Cs” (confidence, convenience, complacency) and “5Cs” (confidence, convenience, complacency, calculation, collective responsibility) models exploring vaccine hesitancy [4,43]: disbelief in effectiveness, fear of side effects, mistrust in institutions and policy practices (“confidence”), low susceptibility to disease (“complacency”), negligence (“convenience”), absence of health professionals’ recommendation and insufficient information (“calculation”). In line with previous studies, doubting the effectiveness of vaccines acts as a major deterrent in curbing immunization efforts [34,35,44,45,46,47,48], as is participants’ belief in good health and thus low perceived risk of vaccine-preventable diseases [34,44,45,46,47,48,49,50]. Lack of advice, endorsement and guidance by health professionals reduces vaccine uptake [35,41,48,50,51,52], as is insufficient information about preventable diseases and the respective vaccines [46,47,51,52]. Our participants highlighted negligence as the root of no uptake and committed to prompt immunization, an argument also expressed in previous studies [41,46]. Additionally, two themes uniquely related to COVID-19 vaccines were spotlighted in our study. Though concern over adverse outcomes of vaccination is a finding regularly reported in the literature [34,44,45,47,48,49], our participants were concerned solely about the adverse effects of the COVID-19 vaccine. This fear affected the public’s adherence to preventive measures against the pandemic, hampering vaccine uptake [41]. Factors such as mistrust in governments, institutions and vaccination policies have always driven vaccine skepticism, viewed as a form of protest [44,46]. Yet, the pandemic amplified perceived dubiousness and mistrust in authorities and played a pivotal role in the development of hesitancy [13]. Our results are congruent with pre-pandemic studies in Greece, which had also reported on important barriers regarding adult vaccine hesitancy; negative experience from former vaccinations, fear of needles, suspicion, and skepticism towards the pharmaceutical industry, as well as accessibility barriers, such as transportation to the practice/pharmacy/vaccination center could influence health behaviors [16].

### 4.3. Strengths and Limitations

To the best of our knowledge, this is the first study in Greece examining the attitudes towards vaccines proposed by the National Adult Immunization Program, as well as towards COVID-19, in the aftermath of the pandemic. This allows for comparison of coverage, awareness, and concerns in the Greek population with previous studies as well as investigation of attitudes towards COVID-19 vaccines. Its mixed methodology allowed us to go into greater depth, quantitatively approach participants’ attitudes with a valid and reliable scale and explore the stances of a larger population compared to traditional, small-sample qualitative studies.

Our study has a few limitations. It is geographically limited to specific communities in western Macedonia that may not allow for the generalization of findings. However, the age distribution of our sample can be considered a very good approximation of the age distribution of the Greek general population [53]; 18–29 years (11.9% in our sample vs. 17.7% in Greek population), 30–39 years (15.0% vs. 18.3%), 40–49 years (18.2% vs. 17.7%), 50–59 years (22.5% vs. 16.0%), 60–64 years (11.1% vs. 7.01%) and 65+ years (21.3% vs. 23.6%). In terms of sex representation in our sample, male sex was only slightly underrepresented compared to the reference population (41.5% vs. 48.9%). As a result, age- and sex-standardized vaccination rates were not calculated. Furthermore, as data were self-reported, recall errors might have led to underestimation or overestimation of vaccination rates, particularly among older participants who might not accurately recall their vaccination status. However, the use of such data appears to be an acceptable method in similar studies examining vaccination status [54]. Additionally, adults might not have been able to accurately recall adolescent vaccinations and therefore tetanus coverage might have been underestimated. Although participation was voluntary and vaccine advocates were more likely to accept participation, response rate amongst randomly selected customers was high.

## 5. Conclusions

Although participants hold affirming stances towards adult vaccination, coverage for three out of the four vaccines recommended by the National Adult Immunization Program is suboptimal. Vaccination coverage for influenza and COVID-19 was reportedly high among high-risk groups, while only two-thirds of those in high-risk groups for pneumococcal disease were vaccinated. Coverage rates for tetanus and herpes zoster were the lowest. The multivariate logistic regression analysis highlighted the importance of attitudes towards the value of adult vaccination and the influence of healthcare professionals’ strong recommendation in determining adult vaccination uptake. Qualitative research identified common themes behind vaccine hesitancy. The uptake of COVID-19 vaccine seems to have followed a significantly different pattern in terms of predicting factors compared to all the other studied vaccines and this was also revealed in the qualitative analysis where fear of adverse effects and distrust in authorities were solely reported for this vaccine. Doubts even in vaccine supporters, high ignorance rates and participants’ wish for more information in the National Adult Immunization Program illuminate the need for systematic interventions to increase uptake and coverage. Successful strategies can only be built on understanding the beliefs, fears and expectations regarding diseases and vaccines. Public health communication and policy should utilize trust in health professionals to communicate the importance, offer personalized approaches and promote primary prevention in the post-pandemic era.

## Figures and Tables

**Table 1:** Sociodemographic characteristics of study participants (n = 395).

Sample Characteristics	n (%)
**Pharmacy**	
A (Amyntaio)	180 (45.6%)
B (Amyntaio)	80 (20.3%)
C (Amyntaio)	45 (11.4%)
D (Levaia)	90 (22.7%)
**Sex**	
Men	164 (41.5%)
Women	231 (58.5%)
**Age groups (years)**	
18–29	47 (11.9%)
30–39	59 (15%)
40–49	72 (18.2%)
50–59	89 (22.5%)
60–64	44 (11.1%)
65+	84 (21.3%)
Educational background *	
Elementary school	57 (14.5%)
Junior high school	33 (8.4%)
High school	112 (28.4%)
Vocational training institute	50 (12.7%)
University/technological educational institute	110 (27.8%)
MSc	32 (8.1%)
Employment status *	
Employed	234 (59.4%)
Unemployed	51 (12.9%)
Student	13 (3.3%)
Retired	96 (24.4%)
**Marital status**	
Married	269 (68.8%)
Single	86 (21.8%)
Widow(er)	29 (7.3%)
Divorced	11 (2.8%)
Underage children **	
Yes	120 (30.4%)

* n = 394 valid responses, ** n = 390 valid responses.

**Table 2:** Participants’ medical history, smoking status and reason for attending the pharmacy (n = 395).

Sample Characteristics	n (%)
**Current smoker**	
Yes	103 (26.5%)
**Chronic health conditions**	
Hypertension	138 (34.9%)
Asthma	19 (4.8%)
Diabetes mellitus	60 (15.2%)
Coronary artery disease	31 (7.8%)
Heart failure	11 (2.8%)
Malignancy	12 (3%)
Thyroid conditions	61 (15.4%)
Prostate diseases	28 (7.1%)
Hypercholesterolemia	89 (22.5%)
Autoimmune diseases	7 (1.8%)
Other	61 (15.4%)
**Reason for attending the pharmacy**	
Chronic medication	140 (35.4%)
Urgent medication	128 (32.4%)
Other *	127 (32.2%)

* Cosmetics, baby care products, nutritional supplements, tests (i.e., pregnancy, SARS-CoV-2 rapid antigen test), etc.

**Table 3:** Mean scores of ATAVAC scale and subscales for each one of the five participant categories according to their responses in the continuum of vaccine hesitancy.

Mean Score per Category	Category in the Continuum of Vaccine Hesitancy				
	Accept All Adult Vaccines	Accept Adult Vaccines, but Unsure	Accept Some Adult Vaccines, but Delay and Refuse Some	Refuse Adult Vaccines, but Unsure	Refuse All Adult Vaccines
ATAVAC scale	5	4.54	4	3.27	2.81
Value of adult vaccination subscale	5.28	4.87	4	2.85	2.42
Safety concerns subscale	3.5	2.5	2	2	2
Perceived barriers subscale	5.5	5.5	5	5	5

**Table 4:** ATAVAC results per item (mean, SD, percentages of valid responses and absolute numbers after combining those falling into negative categories).

ATAVAC Item	Mean (SD)	“Disagree” and “Strongly Disagree” n (%)
I fear the immediate complications of a vaccine (such as allergic reactions)	3 (1.5)	87/384 (22.7%)
I fear the potential impact of vaccines on my health in the future	3.23 (1.5)	106/383 (27.7%)
I believe in the value of vaccination	5.14 (1)	14/381 (3.7%)
It is difficult for me to access the doctor for vaccination (I cannot find an appointment, or the office is too far away or there is no transportation, etc.)	5.3 (0.9)	343/382 (89.8%)
I believe that vaccines are necessary for adults	4.97 (1.1)	15/388 (3.9%)
I believe that the benefits of vaccination outweigh the potential risks	4.88 (1.1)	17/389 (4.4%)
I think if I get ill, I will get more antibodies (better body auto-defense) than if I just get a vaccination	3.87 (1.4)	153/386 (39.7%)
I believe that vaccines are very effective in protecting me from getting a disease	4.52 (1)	19/393 (4.8%)
I haven’t had a vaccine as an adult so far, so I don’t need it	5.27 (1.1)	335/382 (87.7%)
I believe that vaccines should only be given to children	5.32 (0.9)	343/384 (89.3%)
I have financial difficulty in paying for a visit to a doctor or I can’t afford the transportation costs to the office to have the vaccines I need	5.14 (1)	323/384 (84.1%)

**Table 5:** Vaccination coverage for seasonal influenza, tetanus, pneumococcal disease, herpes zoster and COVID-19, per age group and sex.

Age Group	Influenza n (%)	Tetanus n (%)	Pneumococcal Disease n (%)	Herpes Zoster n (%)	COVID-19 1 Dose * n (%)	COVID-19 2 Doses ** n (%)	COVID-19 ≥3 Doses *** n (%)
**All age groups**							
Total	189/394 (48%)	130/393 (33.1%)	105/394 (26.6%)	33/395 (8.3%)	7/394 (1.8%)	45/394 (11.4%)	295/394 (74.8%)
Men	85/164 (51.8%)	73/163 (44.8%)	52/163 (31.9%)	17/164 (10.4%)	3/164 (1.8%)	14/164 (8.5%)	134/164 (81.7%)
Women	104/230 (45.2%)	57/230 (24.8%)	53/231 (22.9%)	16/231 (6.9%)	4/230 (1.7%)	31/230 (13.5%)	161/230 (70%)
**18–29 years old**							
Total	7/47 (14.9%)	26/47 (55.3%)	3/47 (6.4%)	-	1/46 (2.2%)	14/46 (30.4%)	19/46 (41.3%)
Men	1/17 (5.9%)	14/17 (82.3%)	0/17 (0%)	-	0/17 (0%)	4/17 (23.5%)	10/17 (58.8%)
Women	6/30 (20%)	12/30 (40%)	3/30 (10%)	-	1/29 (3.4%)	10/29 (34.5%)	9/29 (31%)
**30–39 years old**							
Total	12/59 (20.3%)	17/59 (28.8%)	5/59 (8.5%)	-	1/59 (1.7%)	6/59 (10.2%)	45/59 (76.3%)
Men	3/16 (27.1%)	9/16 (56.2%)	2/16 (12.5%)	-	1/16 (6.2%)	0/16 (0%)	15/16 (93.7%)
Women	9/43 (20.9%)	8/43 (18.6%)	3/43 (6.7%)	-	0/43 (0%)	6/43 (14%)	30/43 (69.8%)
**40–49 years old**							
Total	29/71 (40.85%)	26/70 (37.1%)	9/71 (12.7%)	-	4/72 (5.6%)	11/72 (15.3%)	50/72 (69.4%)
Men	10/26 (38.5%)	9/25 (36%)	5/25 (20%)	-	2/26 (7.7%)	3/26 (11.5%)	18/26 (69.2%)
Women	19/45 (42.2%)	17/45 (37.8%)	4/46 (8.7%)	-	2/46 (4.3%)	8/46 (17.4%)	32/46 (69.6%)
**50–59 years old**							
Total	41/89 (46%)	26/89 (29.2%)	18/89 (20.2%)	-	0/89 (0%)	9/89 (10.1%)	65/89 (73%)
Men	15/32 (46.9%)	13/32 (40.6%)	6/32 (18.7%)	-	0/32 (0%)	2/32 (6.3%)	24/32 (75%)
Women	26/57 (45.6%)	13/57 (22.8%)	12/57 (21%)	-	0/57 (0%)	7/57 (12.3%)	41/57 (71.9%)
**60–64 years old**							
Total	28/44 (63.6%)	15/44 (34.1%)	16/44 (36.4%)	8/44 (18.2%)	0/44 (0%)	1/44 (2.3%)	41/44 (93.2%)
Men	11/21 (52.4%)	10/21 (47.6%)	7/21 (33.3%)	3/21 (14.3%)	0/21 (0%)	1/21 (4.8%)	20/21 (95.2%)
Women	17/23 (73.9%)	5/23 (21.7%)	9/23 (39.1%)	5/23 (21.7%)	0/23 (0%)	0/23 (0%)	21/23 (91.3%)
**65+ years old**							
Total	72/84 (85.7%)	20/84 (23.8%)	54/84 (64.3%)	25/84 (29.8%)	1/84 (1.2%)	4/84 (4.8%)	75/84 (89.3%)
Men	45/52 (86.5%)	18/52 (34.6%)	32/52 (61.5%)	14/52 (26.9%)	0/52 (0%)	4/52 (7.7%)	47/52 (90.4%)
Women	27/32 (84.4%)	2/32 (6.2%)	22/32 (68.7%)	11/32 (34.4%)	1/32 (3.1%)	0/32 (0%)	28/32 (87.5%)

* 1 dose indicates partial/incomplete vaccination; ** 2 doses indicate complete primary COVID-19 vaccination; *** ≥3 doses indicate booster shots.

**Table 6:** Source of information that mostly influenced participants’ views regarding each vaccine.

**Source**	**Influenza** **n (%)**	**Tetanus ** **n (%)**	**Pneumococcal Disease** **n (%)**	**Herpes Zoster** **n (%)**	**COVID-19** **n (%)**
Family physician	153/394 (38.8%)	86/393 (21.9%)	105/394 (26.6%)	81/395 (20.5%)	198/392 (50.5%)
Specialists	59/393 (15%)	70/393 (17.8%)	49/394 (12.4%)	31/395 (7.8%)	105/395 (26.6%)
Pharmacist	33/394 (8.4%)	20/393 (5.1%)	21/394 (5.3%)	22/394 (5.6%)	53/395 (13.4%)
Friends/family	22/394 (5.6%)	6/393 (1.5%)	10/394 (2.5%)	23/395 (5.8%)	36/395 (9.1%)
Mass media	30/393 (7.6%)	19/392 (4.8%)	24/394 (6.1%)	45/395 (11.4%)	59/395 (14.9%)
Internet	27/393 (6.9%)	24/393 (6.1%)	23/394 (5.8%)	40/394 (10.2%)	47/395 (11.9%)
I am not sufficiently informed about this vaccine	99/394 (25.1%)	165/393 (42%)	173/393 (44%)	169/395 (42.8%)	7/395 (1.8%)

**Table 7:** Illustrative quotes from the open-ended question exploring the reasons for not getting vaccinated against each disease, allocated in overarching themes. Participants’ number, sex and approximate age are presented for each quote.

**Themes**	**Influenza**	**Tetanus**	**Pneumococcal Disease**	**Herpes Zoster**	**COVID-19**
Absence of health professionals’ recommendation	*“My doctor didn’t recommend it.”* P381, woman, late 50s	*“None recommended it to me.” * P96, woman, late 30s	*“My doctor didn’t recommend it.” * P94, woman, early 70s	*“It was not recommended.” * P59, man, mid-60s	
Perception of low susceptibility to disease	*“I just turned 60 and until now I don’t get easily sick, not even from influenza.”* P1, man, early 60s *“I believe there is no need since I feel good about my health.” * P122, man, early 60s	*“I didn’t have any injuries.” * P31, woman, early 50s *“I don’t consider it necessary.” * P72, man, early 40s *“I didn’t hurt/cut myself to seek aid.”* P268, woman, early 50s	*“I believe there is no need since I feel good about my health.” * P122, man, early 60s *“I don’t want to do it yet.” * P181, man, mid-60s *“There was no need to vaccinate.” * P241, man, late 60s	*“I think I don’t need this.” * P252, woman, mid-60s *“As I contracted the disease in the past, I think I won’t get sick again.” * P282, man, early 60s	*“I don’t belong in a vulnerable group.” * P107, woman, early 30s *“I think it’s just like the flu.” * P134, man, mid-50s *“I believe in natural immunity through disease.”* P224, man, mid-30s
Dispute of vaccine effectiveness	*“I am against adult vaccination. I believe you get better immunity after contracting a disease.”* P50, man, early 40s *“I didn’t consider it necessary, as this vaccine doesn’t protect you from all variants.” * P202, woman, mid-30s *“I don’t think the vaccine will protect me.” * P338, man, late 50s	*“I don’t believe in the effectiveness of any vaccine.” * P50, man, early 40s	*“I don’t trust it.” * P175, woman, early 70s		*“I don’t think it’s necessary because of my age and because after a few months they cease protection, so they’re not effective enough.” * P26, woman, late 20s *“I don’t trust it.” * P35, woman, mid-30s *“Because I think this vaccine is a lie/mockery.”* P50, man, early 40s *“I didn’t want to do it because it’s new and I don’t trust its effectiveness.” * P118, man, late 50s *“Not enough research was done.” * P119, woman, early 40s
Negligence	*“I did it last year and [this year] I neglected it.”* P88, woman, late 30s	*“Negligence.” * P3, woman, early 50s	*“I neglected it. I will do it.” * P287, man, early 70s	*“Negligence” * P274, woman, mid-60s	
Insufficient information (regarding disease and/or vaccine)		*“I am not informed.” * P8, woman, mid-70s *“I didn’t know it was needed.” * P12, woman, late 30s *“I did it in my youth. I didn’t know that it should be repeated.”* P280, man, late 60s *“I am not aware of this disease.” * P116, man, early 80s *“I don’t know what tetanus is.”* P212, woman, early 60s	*“I don’t know it.” * P37 woman, mid-60s *“I was never told about the second dose.”* P58, woman, mid-60s *“I didn’t know I had to do it.”* P103, man, early 70s	*“I am not informed.” * P103, man, early 70s *“I had no information about it.”* P331, man, mid-60s	
Distrust in authorities/opposition to obligatory vaccination					*“They [authorities] lie.” * P73, man, late 50s *“I am against any obligation.” * P4, woman, late 20s
Fear of side effects					*“I’m afraid of the immediate and the future side effects.” * P60, woman, early 30s *“It is in an experimental stage, there are side effects and that’s why I’m afraid.”* P83, woman, mid-50s *“I’m afraid of side effects, e.g., thrombosis, and that I might have an allergy.”* P269, woman, early 60s *“I consider it dangerous for health.” * P50, man, 43 years old

**Table 8:** Factors associated with influenza, tetanus, pneumococcal, herpes zoster and COVID-19 vaccine uptake.

Variables	Influenza Vaccine		Tetanus Vaccine		Pneumococcal Vaccine		Herpes Zoster Vaccine		COVID-19 Vaccine	
	aOR * (95% CI)	*p*-Value	aOR * (95% CI)	*p*-Value	aOR * (95% CI)	*p*-Value	aOR * (95% CI)	*p*-Value	aOR * (95% CI)	*p*-Value
**Sociodemographic ** **factors**										
Age	**1.04** (1.01–1.06)	0.017	**0.97** (0.95–1.00)	0.044	**1.05** (1.01–1.08)	0.005	**1.05** (1.00–1.10)	0.043	1.02 (0.98–1.06)	0.380
Males vs. females	0.94 (0.50–1.76)	0.847	**2.79** (1.50–5.19)	0.001	1.38 (0.68–2.80)	0.370	1.21 (0.46–3.15)	0.704	1.18 (0.48–2.90)	0.716
Married vs. other	1.14 (0.58–2.24)	0.706	0.59 (0.30–1.18)	0.135	0.51 (0.23–1.15)	0.104	**0.16** (0.05–0.56)	0.004	1.75 (0.66–4.65)	0.265
Low educational level	0.56 (0.20–1.56)	0.264	1.68 (0.60–4.71)	0.326	0.78 (0.26–2.35)	0.665	0.727 (0.19–2.77)	0.640	0.52 (0.10–2.81)	0.451
**Health status factors**										
Smoking	0.60 (0.30–1.19)	0.141	0.96 (0.50–1.85)	0.91	0.75 (0.33–1.68)	0.478	**0.15** (0.03–0.69)	0.015	1.33 (0.49–3.61)	0.572
Chronic diseases	**2.77** (1.34–5.70)	0.006	1.47 (0.69–3.11)	0.319	**3.03** (1.21–7.57)	0.018	**9.32** (1.77–49.11)	0.008	0.83 (0.29–2.43)	0.737
**ATAVAC subscales**										
Value of adult vaccination	**2.45** (1.50–4.01)	<0.001	1.17 (0.74–1.84)	0.510	1.52 (0.84–2.75)	0.168	**3.24** (1.31–8.04)	0.011	**5.75** (3.03–10.93)	<0.001
Safety concerns	1.19 (0.94–1.50)	0.156	1.11 (0.87–1.41)	0.423	1.04 (0.80–1.37)	0.756	0.83 (0.58–1.20)	0.321	1.51 (0.97–2.34)	0.070
Perceived barriers	0.82 (0.55–1.20)	0.306	**1.54** (1.03–2.32)	0.036	1.08 (0.71–1.65)	0.718	1.38 (0.82–2.33)	0.231	0.85 (0.51–1.41)	0.523
**Doctor’s recommendation**	**11.33** (5.86–21.90)	<0.001	**22.82** (11.80–44.14)	<0.001	**32.41** (13.63–77.08)	<0.001	**37.66** (10.02–141.56)	<0.001	1.78 (0.69–4.56)	0.232
**Pharmacist’s recommendation**	**5.25** (1.79–15.42)	0.003	**4.01** (1.13–14.24)	0.032	**8.43** (2.21–32.11)	0.002	**19.52** (3.11–122.72)	0.002	0.63 (0.20–1.95)	0.425
**Mass media campaign**	0.73 (0.22–2.41)	0.604	1.48 (0.33–6.63)	0.608	1.10 (0.16–7.39)	0.926	0 (0.00–0.000)	0.992	0.45 (0.17–1.21)	0.115

* Adjusted odds ratio.

## Data Availability

The data presented in this study are available on request from the corresponding author.
